# Health workers’ perspectives on self-monitoring of blood pressure by pregnant women: a qualitative study among community health workers, midwives, doctors and health system managers in Lombok, Indonesia

**DOI:** 10.1136/bmjgh-2024-017532

**Published:** 2025-03-22

**Authors:** Tigest Tamrat, Yuni Dwi Setiyawati, Raissa Manika Purwaningtias, Nya Jeumpa Madani, María Barreix, Antoine Geissbuhler, Anuraj H Shankar, Özge Tunçalp

**Affiliations:** 1 UNDP/UNFPA/UNICEF/WHO/World Bank Special Programme of Research, Development and Research Training in Human Reproduction (HRP),Department of Sexual and Reproductive Health and Research, World Health Organization, Geneve, Switzerland; 2 University of Geneva Faculty of Medicine, Geneva, Switzerland; 3 Summit Institute for Development, Mataram, Indonesia; 4 Department of Radiology and Medical Informatics, University of Geneva Faculty of Medicine, Geneva, Switzerland; 5 Nuffield Department of Medicine, University of Oxford, Oxford, UK; 6 Clinical Research Unit, University of Oxford, Jakarta, Indonesia

**Keywords:** Global Health, Health education and promotion, Health services research, Maternal health, Public Health

## Abstract

**Introduction:**

Hypertensive disorders of pregnancy (HDPs) are a leading cause of maternal mortality and morbidity globally but could be mitigated through accurate and timely blood pressure (BP) monitoring. Self-monitoring of blood pressure (SMBP) in pregnancy is an emerging approach for the management of HDPs but mainly studied from the perspectives of pregnant women, in high-income contexts, or tertiary care. This qualitative study explored health workers’ perspectives on SMBP by pregnant women, including through using a smartphone application, within primary healthcare settings of Lombok, Indonesia.

**Methods:**

We conducted focus group discussions (FGDs) and in-depth interviews (IDIs) with community health workers, community-based midwives, facility-based midwives from primary healthcare centres, obstetrician/gynaecologists and health system managers (including heads of facilities, midwife supervisor and District Health Office administrators). Themes were grouped according to the normalisation process theory, which includes (1) coherence/understanding of the intervention, (2) cognitive participation/perceived value and (3) collective action/how the intervention will affect processes and organisational practices.

**Results:**

We recruited 68 participants across 8 FGDs and 26 IDIs. SMBP was perceived to improve timeliness of care and detection of high BP, but health workers expressed concerns about women’s ability to accurately self-monitor, reliability and affordability of BP devices, and accountability and management of SMBP results. Embedding SMBP into routine antenatal care counselling, collaboration with family members, clarification on liability implications and protocols on SMBP follow-up actions, and use of digital communication channels were cited as potential ways to facilitate uptake of SMBP.

**Conclusion:**

For SMBP to be valued by health workers and effectively integrated into the health system as a strategy for addressing HDPs, it needs to be accompanied by clear clinical and data management protocols, referral mechanisms, reassurance on the accuracy and trust in the self-monitored measurements and demonstration of timeliness in the provision of follow-up care for pregnant women.

**WHAT IS ALREADY KNOWN ON THIS TOPIC**

- Self-monitoring of blood pressure (SMBP) by pregnant women is an emerging approach for the management of hypertensive disorders of pregnancy. The majority of this research is from the lens of pregnant women, which is critical but also needs to be complemented by health workers’ perspectives for SMBP to be effectively integrated into health systems.
- Prior research on health workers’ views has primarily been conducted among tertiary care providers or in high-income settings. These studies indicate health workers’ willingness to support SMBP by pregnant women but concerns surrounding liability and lack of clarity in clinical management protocols.

**WHAT THIS STUDY ADDS**

- The study was conducted across a diverse set of health worker cadres at different levels of the health system, including community health workers, village-based and health facility-based midwives, obstetricians/gynaecologists and district managers within primary healthcare catchment areas of Indonesia.
- The use of a smartphone BP measurement application (OptiBP) was also explored for potentially addressing challenges related to access of BP measurement devices.

**HOW THIS STUDY MIGHT AFFECT RESEARCH, PRACTICE OR POLICY**

- The study identified key considerations for SMBP to be valued by health workers and institutionalised as a strategy for addressing HDP. This includes the need for clinical and data management protocols, communication of workflow and liability implications, reassurance on the accuracy and trust in the self-monitored measurements and demonstration of timeliness in the provision of follow-up care for pregnant women.
- Health workers cited mechanisms for bolstering SMBP by pregnant women, such as integration of SMBP into routine antenatal care counselling, involvement of family members and use of digital communication channels such as WhatsApp.

## Introduction

 Hypertensive disorders of pregnancy (HDPs) are one of the leading causes of maternal mortality and morbidity globally. They comprise (1) chronic hypertension, (2) gestational hypertension (hypertension identified in the latter half of pregnancy), (3) pre-eclampsia-eclampsia, which consists of gestational hypertension and proteinuria after 20 weeks of gestation, and (3) pre-eclampsia superimposed on chronic hypertension.^1^,^5^ Routine blood pressure (BP) monitoring in pregnancy is critical for the appropriate management of HDPs and is often conducted by a healthcare provider during antenatal care (ANC).^6 7^ To supplement BP monitoring by health workers and facilitate timely detection of high BP, the WHO also recommends that pregnant women with HDPs can self-monitor their blood pressure (SMBP).^8 9^ However, knowledge gaps remain on the feasibility and implementation considerations for SMBP by pregnant women in low- and middle-income contexts (LMICs).^8^

SMBP in pregnancy has primarily been conducted in high-income settings, such as the UK, North America and Australia,^10^,^22^ and was particularly accelerated due to the COVID-19 pandemic which necessitated mechanisms for continued BP monitoring outside of health facilities.^23 24^ Efforts are now emerging to expand SMBP to settings with a high burden of HDPs, such as Africa and Asia.^25^,^28^ While most of this research on SMBP justifiably focuses on the needs of pregnant women, the perspectives of the health workers and managers cannot be overlooked when integrating this self-care intervention into the health system. Moreover, health workers play a critical role in ensuring appropriate management of care and are instrumental in the socialization of SMBP.

Research on health workers’ perspectives on SMBP predominantly focuses on the general hypertensive population. These studies indicate clinicians’ willingness to adopt SMBP, particularly as it brings the opportunity to differentiate self-monitored BP values from ‘white coat’ hypertension.^29^,^32^ However, health workers have also cited the uncertainty of care protocols and the ramifications of SMBP on medication management.^32^ A fewer set of studies have examined health workers’ views on the introduction of SMBP by pregnant women or within the context of ANC, where there are additional considerations, such as the implications of newly diagnosed hypertension on both the fetus and the pregnant woman.^16 22 27 28^ In two studies conducted in the UK, health workers expressed the willingness to share responsibility with pregnant women for SMBP but also sought clarifications on mechanisms to integrate this intervention across the maternal health continuum of care.^16 22^ Similarly, obstetricians in Ghana found SMBP by pregnant women to be potentially beneficial in reducing delays in care^27 28^ but also noted significant barriers, such as the availability of BP devices, lack of communication pathways, and low health literacy.^27^

This qualitative study examined health workers’ and health system managers’ perspectives towards SMBP to inform considerations for integrating SMBP in pregnancy within the primary healthcare settings of Lombok, Indonesia. The study complements perspectives from pregnant women in an effort to broaden the evidence base on SMBP, particularly as available studies conducted in LMICs have focused primarily on tertiary care providers.^26^,^2833^ As a secondary objective, the study also explored perspectives on using a smartphone BP measurement application (OptiBP)^34^,^38^ for possibly addressing the challenges identified in the literature related to access to and use of BP measurement devices. The OptiBP application functions by using a smartphone camera to record photoplethysmographic optical pulse waves derived from blood volume changes at the fingertips and estimate BP values using an algorithm.^37 39^

## Methods

### Study design

We conducted qualitative research consisting of focus group discussions (FGDs) and in-depth interviews (IDIs) to examine health workers’ perspectives, including their perceptions and attitudes, towards SMBP by pregnant women. To gain a comprehensive overview of health system actors, the study was conducted across different health worker cadres, including community health workers (CHWs), village-based and health facility-based midwives, obstetricians/gynaecologists and health system managers (including heads of facilities, midwife supervisor and District Health Office (DHO) administrators). The study was conducted in West Lombok Regency, Lombok Island of Indonesia, across the catchment area of six primary healthcare facilities (Lingsar, Narmada, Penimbung, Meninting, Jembatan Kembar and Gerung) and two referral hospitals (Patut Patuh Patju and Awet Muda). This location was selected as it builds on a prior validation study of the OptiBP smartphone application and a complementary assessment on perceptions and attitudes of pregnant women towards SMBP.^40 41^ Furthermore, these studies were conducted under the auspices of the Summit Institute for Development (SID), an Indonesian research and development organization based in Lombok leading broader research on digital health interventions to strengthen primary health care.

### Sampling

The study included a range of health workers and managers as described in table 1. To reduce heterogeneity in the FGDs, CHWs were separated based on their sex and age group, defined as being older or younger than the median. Similarly, midwives were assigned to separate FGDs based on whether they were based in a community health post or health centre/facility and stratified into older and younger groups based on the median age. The sample size was based on estimates to achieve thematic saturation and availability of participants in categories, such as health system managers and doctors.

**Table 1:** Study participants by type and method

Method	Female CHWs (n=16)	Male CHWs (n=13)	Community-based midwives (n=16)	Facility-based midwives (n=14)	Obstetrician/gynaecologist (n=4)	Health system managers *(n=5)
FGDs	2 FGDs of 8 participants	2 FGDs of 6–7 participants	2 FGDs of 8 participants	2 FGDs of 7 participants	N/A	N/A
IDIs	4 (selected from FGDs)	4 (selected from FGDs)	4 (selected from FGDs)	5 (selected from FGDs)	4	5

* This included one coordinator of the family health section of the West Lombok DHO; two midwife coordinators and two heads of primary health centres. This includes representatives from the District Health Office; midwife coordinators; and heads of primary health centres.

CHWscommunity health workersFGDsfocus group discussionsIDIsin-depth interviews

Midwives and CHWs were identified through the DHO and facility networks under its hierarchical systems, which are Public Health Centres (Puskesmas) and Maternity Clinics (Polindes). Following the identification and approval from the DHO, the study team randomly selected eligible health workers and invited them to an information session on the study and obtained informed consent. In the case of CHWs where there are both male and female cadres, FGD participants were grouped based on sex and age as either younger or older than the median age for that participant type.

Obstetricians/gynaecologists were recruited from the two referral hospitals in West Lombok. We sent a letter to these hospitals and invited five obstetricians/gynaecologists and received confirmation from four of them to attend IDIs. Health system managers were invited as key informants; this group consisted of midwife coordinators/supervisors, heads of the primary healthcare centres and the unit head from the DHO.

FGDs were conducted with CHWs and midwives, and a subset of these participants who expressed divergent views were selected for a subsequent IDI for further exploration. We employed both qualitative methodologies to first identify emerging findings through FGDs, which were examined for better understanding in greater detail through IDIs.^42^,^45^ Where there were five or fewer participants per cadre, as in the case of health system managers and obstetricians/gynaecologists, only IDIs were conducted.

### Procedures

FGDs and IDIs were conducted in April and May 2023 in a hotel meeting room in Mataram, Lombok. The study team scheduled interviews based on a mutually agreed time during the workday. Transportation and meal packages were provided to all participants. Interviews were conducted in Bahasa by SID research staff. Interviewers had a public health background and were trained on the interview guide; one of the interviewers was a non-practising midwife and lecturer. With the exception of interviews with male CHWs, all other interviews were conducted by female researchers.

Semistructured interview guides for the FGDs and IDIs were developed in English and subsequently translated into Bahasa Indonesian. As the SMBP intervention is to be done by pregnant women, we asked health workers their views on (1) experiences in providing ANC, (2) self-managing of pregnancy in general, (3) SMBP by pregnant women and (4) data flow and health system linkages to facilitate SMBP. Prompts for probing were included as part of the interview guide. The smartphone BP application was introduced during the interviews to obtain health workers’ initial feedback but was not used by participants outside of the interviews. There were no individuals present besides the participants and researchers during the interviews.

### Data analysis

Each audio recording was de-identified and double transcribed. The two versions of the Bahasa Indonesian transcripts were cross-checked for quality assurance, and discrepancies were resolved based on the original recording. Each revised transcript was subsequently translated by two independent and professional translators, in which the research team compared the files and reconciled differences to yield the final transcripts for analysis. Transcripts were not returned to the study participants. Interviewers recorded field notes and shared reflections on weekly calls with the broader research team.

We analysed the transcripts using the Ritchie and Lewis (2013) framework for qualitative data management, which consists of (1) familiarisation and identification of initial themes, (2) labelling or tagging the data and (3) summarising or synthesising the data.^46^ A member of the research team (TT) read a subset of the translated transcripts to identify themes applying both inductive reasoning based on emerging patterns from the data and deductive analysis linked to themes identified in the other studies.^16 30 32^ Using the approach of the BUMP study for evaluating the perspectives of health workers, the initial set of codes were developed using the normalisation process theory (NPT).^47^ The NPT framework is based on the theory of innovation diffusion and addresses factors needed for successful implementation and integration of new or complex interventions into routine work (normalisation).^47^ We used three of the four domains from the NPT framework related to (1) coherence, which pertains to understanding of the intervention; (2) cognitive participation, which links to engagement and perceived value of the intervention; and (3) collective action, which refers to how the intervention will affect organisational processes and practice. The fourth NPT domain of reflexive monitoring was not used as this is assessed once the intervention has been implemented.

After the development of the codebook, interviews were coded independently by TT, NJM and RMP using the Atlas.ti software (Web V.24) for thematic analysis. One of the researchers (TT) coded all transcripts, and the other two researchers (NJM and RS) coded a randomised subset of half of all transcripts. Preliminary findings from the coding and illustrative quotes were reviewed through calls with the broader research team. Illustrative quotes were first selected among the different coders to highlight key findings. These quotes were further reduced in discussion with all coauthors and reviewed to ensure consistency in the analysis and interpretation of findings.

We used the Standards for Reporting Qualitative Research ^48^ and the COnsolidated criteria for REporting Qualitative research checklist^49^ for reporting and adhering to standards in qualitative research.

## Results

The study enrolled 68 participants across 8 FGDs and 26 IDIs (table 2). Study participants generally had at least 1 year of service, with the majority of CHWs and midwives having over 5 years of experience.

**Table 2:** Participant characteristics

Characteristics	Community health workers (n=29)	Community-based midwives (n=16)	Facility-based midwives (n=14)	Obstetrician/gynaecologist (n=4)	Health system managers (n=5)
Sex					
Male	13	0	0	3	2
Female	16	16	14	1	3
Age (years)					
19	2	0	0	0	0
20–25	7	1	0	0	0
26–30	5	1	1	0	0
31–35	3	9	6	1	0
35–40	5	2	1	2	0
41–45	3	1	3	0	1
46+	5	2	3	1	4
Years of service					
<1	4	0	1	1	1
1–5	10	5	5	0	3
5–10	5	1	2	2	0
10+	11	10	6	1	1

There were no male midwives in the study area.

**Figure 1:**
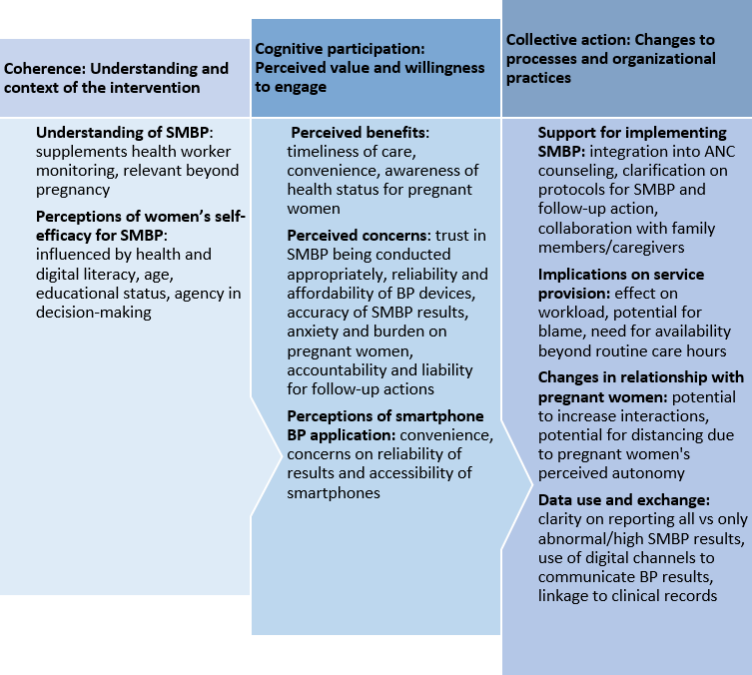
Overview of the themes according to the adapted normalisation process theory.^47^ ANC, antenatal care; BP, blood pressure; SMBP, self-monitoring of blood pressure.

Several themes emerged regarding health workers’ perspectives including: understanding of SMBP, perceptions of pregnant women’s self-efficacy, perceived benefits and concerns, perceptions of the smartphone BP application, support for implementing SMBP, implications of SMBP on service provision, and data use and exchange. These themes were organised according to the NPT^47^ (figure 1) and elaborated in the findings.

### Coherence: understanding of SMBP

The concept of SMBP was generally understood as an important intervention to supplement BP monitoring by health workers. Reasons for its importance included the fluctuating nature of BP and to overcome challenges in accessing health facilities. Some participants, particularly CHWs and health system managers, were interested in expanding SMBP beyond pregnant women, including elderly populations, general populations or those currently living with chronic hypertension. CHWs also demonstrated wanting to be directly involved in SMBP and saw this as an opportunity to increase their skills. However, they also expressed conflations on issues related to BP and haemoglobin, such as requiring ‘blood boosters’ (iron folate supplements) if the BP was low (additional illustrative quotes available in online supplemental file 1).

Yes, it is necessary to do that because blood pressure can fluctuate unpredictably. This is especially important for pregnant women to increase their knowledge about it. And blood pressure, in general, not just for pregnant women, even healthy individuals need to control their blood pressure independently. (IDI, health system manager)

If there is no specific role, meaning if it’s only to monitor the patient’s condition, then the effect is positive … So, we can both know “Oh, it’s like this.” At least we have an idea… For example, their blood pressure is 110/60, but they’re feeling dizzy and have pain in the upper stomach,” then we can say “Oh, okay, let’s see, maybe there’s an error or something,” and we can immediately ask them to go to the health facility. (IDI, community midwife, younger)

Yes, most people who frequently have their blood pressure checked, they will know what it’s going to be like, right. What you have to do is take her to the Community Health Centre. Later they will get a medicine. But for pregnant women, because usually they will get a blood booster if it is low. But if it’s low or high, it’s still given, because it is usually related with low HB [haemoglobin] too, right? (IDI, male CHW, younger)

### Coherence: perceptions of pregnant women’s self-efficacy

Health workers’ perspectives on the feasibility of SMBP were often influenced by how they perceived pregnant women’s background and self-efficacy. Participants mentioned characteristics about pregnant women, such as their educational background, occupation, level of engagement in their health and digital literacy, as factors that could influence SMBP. In addition, health workers noted that pregnant women were not always the sole decision-makers and the role of family and community members in influencing their health-seeking behaviours (additional illustrative quotes available in online supplemental file 1).

For example, if pregnant women are young and have higher education, in my opinion, it’s easy for them. It’s easy because if you look at the way it’s done, it looks easy. But if they are already 35 years old or almost 40, and those in remote areas are a bit difficult, in my opinion. But they need help. (IDI, facility midwife, younger)

They are also not aware that the one responsible for their health is themselves. For them, their body and health depend on their family, and they must seek approval from the decision makers in their family. (FGD, facility midwife, younger)

Yes, for example, if they are supposed to use it regularly but forget. If they are scheduled to self-monitor their blood pressure regularly a certain number of times, they might forget. The first reason was complexity, laziness. Some don't like gadgets, but if someone likes gadgets, they might not have any difficulty. (IDI, health system manager)

### Cognitive participation: perceived benefits of SMBP

SMBP was often perceived as convenient for pregnant women and linked to improving the timeliness of care, including referrals. Midwives, in particular, felt that SMBP could assist them in following up women with HDPs and ensuring appropriate management, while also reducing geographic barriers to accessing health services. Participants also viewed SMBP as a mechanism for increasing awareness among pregnant women about their BP and overall health status (additional illustrative quotes available in online supplemental file 1).

The benefit of this is risk detection by the community itself or by the family itself, so it is very, very good, once they are there, there is a risk, especially for blood pressure, then they are proactive in seeking help so it’s good. (IDI, health system manager)

It's about time because when we encounter such incidents, we have to act quickly in terms of referral systems, so if the pregnant woman already knows her condition, the treatment, and subsequent actions can be initiated more promptly. (FGD, community midwife, older)

For pregnant women, it would make them more aware of their condition. And for the kader (CHW), it would also make their job easier. For example, if there’s a patient and the midwife hasn’t arrived yet, they could ask for help with taking the blood pressure. It would make things easier. But there should be some knowledge first, about how the application works, to make it easier. Then, the numbers displayed on it should be explained so people understand what they mean. (IDI, female CHW, younger)

### Cognitive participation: perceived concerns and barriers to SMBP

Participants cited several barriers and concerns with SMBP, including the reliability and affordability of BP measurement equipment, trust that SMBP would be done correctly and the effect it may have in making pregnant women anxious. Some participants also raised questions about the accuracy of the BP readings and often suggested that measurements would need to be taken again to ascertain the BP levels. Midwives, in particular, mentioned risks of pregnant women over-relying on SMBP and not attending their ANC follow-up. Some participants noted that pregnant women may not have adequate awareness about BP in general or evade SMBP due to financial costs associated with seeking care at a hospital (additional illustrative quotes available in online supplemental file 1).

We are worried that when the results of our examination with the application are different, it will raise doubts. Whether it’s doubts about the application or doubts about the midwife. (FGD, facility midwife, younger)

I think it's helpful, but there are concerns and anxieties from us as midwives. Firstly, it’s about accuracy, and secondly, about the correct usage by the patients themselves…Some patients may become lazy to come directly to the health centre…So, it's not just about self-monitoring all the time. We still need to facilitate them. (IDI, community midwife, younger)

I believe that pregnant women may face some challenges while measuring their own blood pressure at home. This is especially true for those who come from a low socioeconomic background. One of the main challenges is the affordability of the tools required for measuring blood pressure. This includes a blood pressure monitor or a smartphone. Some women may not be able to afford these tools, which may make it difficult to monitor their blood pressure regularly. (IDI, Ob/gyn)

### Cognitive participation: perceptions of using a smartphone-based BP application

Regarding the use of OptiBP, a smartphone, participants noted the convenience of monitoring BP via their mobile phones but also raised concerns about affordability and access to smartphones and internet data. In addition to overall counselling on SMBP, healthcare workers suggested further familiarisation with the functionality of the application and reassurance in the accuracy of the BP readings, including on how sharing phones would affect the BP results. Furthermore, health system managers noted that the smartphone application could primarily serve to gauge BP to be followed by confirmation by a midwife (additional illustrative quotes available in online supplemental file 1).

Geographical location is also an obstacle, some live on top of the mountain too. So, indeed, not all pregnant women are modern and not all have Android phones, even in one family they don't have cell phones. Sometimes they borrow kader’s [CHW’s] cell phones. (FGD, facility midwife, younger)

With just a smartphone, blood pressure can be checked, and the results can be displayed. While the accuracy might not be as reliable as traditional methods, it can serve as a preliminary screening tool. If a pregnant woman receives a reading that indicates something might be abnormal, she can immediately contact a healthcare professional for a more thorough assessment, in my opinion. (IDI, health system manager)

There’s a fear that it might be used by people who don't know the benefits and drawbacks, and they might misuse it. For example, let’s say a family has this device, and a minor in the family uses it… If the reading shows high blood pressure, it could affect the person being tested, and the information might be incomplete or incorrect, not matching the actual condition. (IDI female CHW, younger)

### Collective action: support for implementing SMBP

Although participants mentioned concerns with the introduction of SMBP among pregnant women, they also suggested ways to support pregnant women to effectively self-monitor their BP. Integrating SMBP into the existing ANC health education and counselling, including the home-based record, was highlighted as one of the ways to ensure pregnant women could effectively self-monitor. Midwives cited the use of WhatsApp groups and communication with their family members—such as husbands, parents and children—to bolster SMBP. Health workers highlighted areas to include in the SMBP counselling, such as BP thresholds, familiarisation with the BP measurement device and follow-up actions for women to take in response to their BP results. CHWs also demonstrated a willingness to support pregnant women and serve as a link with the midwives, but they also noted the need to receive adequate training (additional illustrative quotes available in online supplemental file 1).

Health education on this matter can be provided at the Posyandu (Integrated Health Service Post) or during your classes where all pregnant women are gathered. If the information is repeated several times, they will eventually learn. However, if they are left to do it alone, they might not know which steps to take. But, if they are gathered and taught by a competent team, they will understand. For instance, they can be taught during your classes or at the Posyandu [community health post]. (IDI, facility midwife, younger)

I think providing KIE [IEC-Information, Education, Communication) is sufficient. With KIE and the belief that there is no harm in using this device because it actually helps with early screening for mothers, I believe with that understanding, they will use it. Because there is no harm in using this device in terms of, well, we can explain later whether this device has any immediate drawbacks or not, that’s about it… Mandatory education should be provided when the mothers come for their check-ups, including what messages to convey. (IDI, health system manager)

### Collective action: implications on service provision

Participants expressed mixed feelings on how SMBP would change interactions and workload. Generally, they perceived it as something that would support their work but also expressed the need for clarity on communicating SMBP results outside of work hours. Non-managerial healthcare workers, primarily CHWs and community-based midwives, mentioned potential concerns of blame and accountability if something went wrong during the BP monitoring process (additional illustrative quotes available in online supplemental file 1).

Consultation time, we have to be strict with patients who have been referred to this, [that is] the risk. It’s okay, rather than later if something happens, we don't monitor, we will be blamed by the health centre too, why there is no monitoring of pregnant women, and there is a risk, like that. (IDI, community midwife, younger)

As for the workload, not really. The sooner we know, the sooner the patient receives treatment. In fact, the slower we find out, the more our performance is questioned. (IDI, health system manager)

### Collective action: changes in relationship with pregnant women

Participants believed that SMBP may strengthen relationships with pregnant women and the possibility of more frequent contacts due to increased awareness about their health and questions regarding BP. On the other hand, participants also raised concerns that pregnant women may feel less inclined to attend their scheduled ANC contacts due to greater autonomy in managing their health (additional illustrative quotes available in online supplemental file 1).

It [relationship with pregnant women] will be closer, I think. Because if there is a blood pressure device, I won't say that they don't need to come to me. They will probably come to me more often, every time they finish checking their blood pressure. If possible, they can inform me whether it is normal or abnormal. So, our communication will be much more intensive than before. (IDI, community midwife, younger)

Maybe they'd become less motivated to come to the Posyandu [health post] for blood pressure checks. So, if they check it themselves and it seems normal, they might be a bit lazy. (FGD, male CHW, younger)

### Collective action: data use and exchange

Participants reported different aspects on the use and exchange of SMBP data. Health system managers cited the need to integrate SMBP information within other reporting tools, including electronic medical records and similar digital systems for managing pregnancy data. Midwives expressed a greater interest in the use of SMBP information for clinical management and suggested the use of digital communication channels, such as WhatsApp and SMS to communicate SMBP results. Participants presented different perspectives and lack of clarity on the confidentiality of the SMBP data, frequency of data sharing, as well as determining what type of SMBP results should be reported (ie, whether only abnormal/high measurements should be communicated), and storage of SMBP data, such as within a centralised digital system for pregnancy records (additional illustrative quotes available in online supplemental file 1).

Through the phone, maybe the patient can send a photo of the blood pressure result. There are advanced technologies now, right? Sophisticated, they can take a photo of the result and send it through WhatsApp. (IDI, village midwife, older)

In my opinion, if the results are normal and good, there’s probably no need to share them. But when they get abnormal results, it’s better to share them with midwives, at least with the midwife who regularly conducts the examinations and can confirm the results. (IDI, health system manager)

## Discussion

Our research presents critical considerations and perspectives from different cadres of the health workforce in Lombok, Indonesia, on SMBP by pregnant women. Across all participant groups, there was a general assumption that SMBP could increase timeliness in care and early detection of high BP if done with appropriate support. However, participants also raised concerns related to trust and pregnant women’s capabilities to accurately self-monitor, affordability and reliability of BP measurement devices, and communication for follow-up actions. Midwives, in particular, expressed issues regarding trust and repercussions of SMBP if the BP results differed from theirs, or the potential blame if women do not conduct SMBP or seek care appropriately. Considering the central role of midwives in providing ANC, efforts will be critical to address their concerns related to trust and accountability. In contrast, participants who do not work exclusively with pregnant women, such as CHWs and health system managers, demonstrated interest in expanding SMBP to other populations, such as the elderly and hypertensive individuals.

Although participants had concerns about SMBP, midwives, in particular, offered mechanisms to overcome these potential challenges, such as through integrating SMBP into ANC counselling, involving caregivers, using digital communication channels (ie, WhatsApp) to exchange results and information, and establishing clear protocols for managing SMBP results. The study also highlighted limitations and variability in CHWs’ understanding of BP, including perceived linkages between BP and haemoglobin, which would need to be addressed prior to adding the layer of SMBP as part of care during pregnancy.

The exploration into the smartphone BP measurement application provided an opportunity to probe into new ways that SMBP could be made accessible and address the commonly cited challenges of the unavailability of devices.^50 51^ Participants, particularly midwives and health system managers, asserted the need for quality assurance on its reliability, with some noting potential mistrust in digital tools. Health system managers, in particular, also noted equity considerations on access to smartphones and related internet data requirements. As smartphone device capabilities evolve, their use in supporting diagnostics could expand coverage and uptake of SMBP. Furthermore, midwives and CHWs cited other ways digital tools could support SMBP by expediting communication of results and reinforcing counselling for pregnant women on how to self-monitor.

These findings resonate with other studies where health workers found SMBP potentially beneficial for increasing BP awareness and early detection among women with HDPs,^16 21^ but also requested clarity on protocols to address concerns of liability and workload management.^27 28^ The use of the NPT framework, as applied in the BUMP study,^16^ distilled issues related to innovation diffusion, such as the complexity of understanding the innovation, perceived relative advantage and compatibility with values and experiences.^52^ However, this is one of the few studies that explored SMBP among primary healthcare workers, including those based within the community, and set within a low- and middle-income country context.^27 28^ This additional perspective provided insights from community-based midwives and CHWs who work more closely with pregnant women and highlighted additional considerations, such as ways to incorporate SMBP into ANC counselling, implications on workload and notifications outside of regular hours and concerns about women potentially evading SMBP due to the financial implications of seeking care at a referral facility.

A key strength of this study is the diversity of health worker cadres, including managers’ perspectives, which allowed for a comprehensive picture of operational considerations for meaningfully integrating SMBP within the health system and with due consideration to the importance of coordination of care within ANC.^53^ This study also identified key implications for practice that would need to be reflected within SMBP management protocols, such as ensuring appropriate counselling on SMBP to pregnant women; clarifying how, when and to whom pregnant women should communicate SMBP results; establishing a common understanding on liability and actions for follow-up; and defining considerations on confidentiality and storage of BP data, including potential linkages of SMBP data with other digital patient record systems. A limitation of this study is that SMBP has not yet been implemented, which could affect participants’ feedback or reflect social desirability bias. However, this was mitigated through robust sampling and a wide range of included participant categories.

## Conclusion

For SMBP to be valued by health workers and effectively integrated into health systems as a strategy for addressing HDPs, it needs to be accompanied by clear clinical and data management protocols, referral mechanisms, reassurance on the accuracy and trust in the self-monitored measurements, and demonstration of timeliness in the provision of follow-up care for pregnant women. Although these findings offer formative reflections for complementing pregnant women’s perspectives on SMBP,^9^,^1114 19 21 54^ future studies should expand on this work by conducting evaluations once SMBP has been implemented to ensure its impact on ANC service provision and health outcomes.

## supplementary material

10.1136/bmjgh-2024-017532 online supplemental file 1

10.1136/bmjgh-2024-017532 online supplemental file 2

## Data Availability

Data may be obtained from a third party and are not publicly available.
