# Supplementary material for: Health workers’ perspectives on self-monitoring of blood pressure by pregnant women: a qualitative study among community health workers, midwives, doctors and health system managers in Lombok, Indonesia
Source: BMJ Glob Health. 2025 Mar 22;10(3):e017532. doi: 10.1136/bmjgh-2024-017532 (PMC11931923; doi:10.1136/bmjgh-2024-017532)
Supplement: online supplemental file 1 [file bmjgh-10-3-s001.pdf]

Supplement 1: Additional illustrative quotes

Table 1. Additional illustrative quotes for coherence domain

Theme	Illustrative quotes
**Understanding of SMBP**	• *Yes, it is necessary to do that because blood pressure can fluctuate unpredictably. This is especially important for pregnant women to increase their knowledge about it. And blood pressure, in general, not just for pregnant women, even healthy individuals need to control their blood pressure independently.* ***(IDI, Health system manager)***<br><br>• *Actually, about the blood pressure, the elderly needs this more. Not only pregnant women. Elderly are the same. They seldom go to the public health centre if no one accompanies. We know these patients are at risk. At the very least, we should know their blood pressure once a week. That's our challenge… Especially in my area, there are quite a few elderly people. So, it's really important, even for myself, that we monitor them once a week.* ***(FGD, female CHW, older)***
**Perceptions of pregnant women's self-efficacy**	• *Usually, the active ones, even without the equipment, will seek information, curious like that. If they are passive, sometimes we have to remind them, for example, so that it is done or carried out. Maybe that's what makes the difference. It will definitely yield different results.* ***(IDI, community midwife, older)***<br><br>• *They are also not aware that the one responsible for their health is themselves. For them, their body and health depend on their family, and they must seek approval from the decision makers in their family. They will sometimes just keep quiet if the family does not allow them to go to the referral centre. Sometimes they already know that their condition is in danger, but according to the family the patient is in good condition and doesn't need to be referred because if they are referred, they can't do anything.* ***(FGD, facility midwife, younger)***<br><br>• *There are some patients who are proactive and have devices at home. If a patient has a history of high blood pressure before pregnancy, they often measure their blood pressure using digital tools during pregnancy.* ***(IDI, Ob/gyn)***

Table 2. Additional illustrative quotes for cognitive participation domain

Theme	Illustrative quotes
**Perceived benefits of SMBP**	• *Well, according to me, um, it's like this…it makes things easier for them. So, if they have a little complaint, they don't need to immediately hire a motorcycle taxi to visit a healthcare provider. They can do the check-up themselves at home. Now, it depends on each individual, whether they are confident in the results they get from this, what do you call it, the examination results that go through Android. Or maybe if they're not confident, they'll repeat it with the healthcare provider to do the examination.* ***(IDI, facility-based midwife, older)***

Theme	Illustrative quotes
	• *It's about time because when we encounter such incidents, we have to act quickly in terms of referral systems, so if the pregnant woman already knows her condition, the treatment, and subsequent actions can be initiated more promptly.* ***(FGD, community midwife, older)*** <br>• *Uh, it will make it easier. It will make it easier, very helpful. So when they have complaints, and know when it happens, because during this time we never know when their blood pressure rises, we might just say "oh, one month ago it was normal when I reported to the Integrated Health Care", but today it has increased. When did it happen? We don't know…In this digital era, with the help of medical devices, people don't need to take themselves to the health centre to measure their temperature or blood pressure. They also don't need to go to the hospital and wait in line, which sometimes makes people lazy.* ***(IDI, Ob/gyn)***
**Perceived concerns and barriers to SMBP**	• *I think it's helpful, but there are concerns and anxieties from us as midwives. Firstly, it's about accuracy, and secondly, about the correct usage by the patients themselves…Some patients may become lazy to come directly to the health centre… We need to inform patients that even if they monitor their blood pressure at home, it cannot be 100% relied on. They still need to see healthcare professionals. So, it's not just about self-monitoring all the time. We still need to facilitate them.* ***(IDI, community midwife, younger)*** <br>• *If you go to the hospital, apart from being afraid of financial problems, perhaps the biggest problem at the hospital is that it requires a lot of money. So, patients sometimes just surrender to their situation.* ***(IDI, facility midwife, older)*** <br>• *The obstacles that may be encountered are if pregnant women are provided with the equipment, they might be afraid of damage or loss. Secondly, it depends on the pregnant woman's knowledge – whether she understands her condition or not. But of course, we should educate the pregnant woman first about what normal blood pressure is during pregnancy. If there's any new change, she should contact a healthcare worker. Now, it also depends on the pregnant woman's concern for her own condition and the support of her family. For example, if she knows she has high blood pressure, does her husband or family support her in going to the health centre or seeking healthcare? These are the challenges we find.* ***(IDI, health system manager)*** <br>• *That's what I'm afraid of. They don't really understand the problem of blood pressure. At least when checked by the kader (CHW) and go to the Community Health Centre. They've been told it's just normal. they didn't know the normal numbers are like, oh low or high, they don't know.* ***(IDI, male CHW, younger)*** <br>• *Yes, if their blood pressure goes up, they might become anxious. After all, when there's an increase, they can get worried. Pregnant women, if I recall correctly, can get stressed when their blood pressure rises. They start worrying about life and death in those situations. I've experienced it myself. I used to get really stressed, wondering what to do when my blood pressure was too high. Checking it every day is not feasible.* ***(IDI, female CHW, older)***

Table 3. Additional illustrative quotes for collective action domain

Theme	Illustrative quotes
Support for implementing SMBP	• *The important thing is that we educate them first. If their blood pressure rises, they must report it to a healthcare worker. So, if the patient doesn't report it, we must think positively, assuming the patient is fine. However, we still need to be cautious… We need to make sure, since we also have a WhatsApp group for their companions…we can ask the companion if they have been monitoring the patient's blood pressure every day, and whether the results are normal or not. This is because not all pregnant women are honest about their condition.* ***(IDI, facility midwife, older)***<br><br>• *We always emphasize the danger signs that are written in the KIA [home-based record] book because that's the book that they always carry with them. If someone has high blood pressure, for example, they need to take their medicine and come back for follow-up, and they shouldn't ignore symptoms like this, this, and this, but should seek immediate help. We repeat these things over and over again during [counselling] sessions. Because not everyone will understand if we explain it once. We need to repeat it so that they understand.* ***(IDI, village midwife, younger)***<br><br>• *If there are issues or if the results are inconsistent, there should be some connection, at least with the healthcare worker initially. This could change, and eventually, the healthcare worker would provide more clarification for a clearer understanding." (****IDI, female CHW, younger****)*
**Implications on service provision**	• *Well, the concern about adding workload is not there. It's just more about time. If they report frequently, then automatically they will consult with us. However, we may not have as much time as they want because we're racing against the conditions in our health centre and the workload is very high. We start at 8am, with a number of pregnant women in the maternity ward waiting in line, and others waiting for their turn. That's what's worrying. If the context is outside of work, then they may have more freedom to consult. But they don't want to disturb us unless they come to us directly. (****IDI, facility midwife, old****)*<br><br>• *We believe it won't disrupt because the role of healthcare providers is to convey that the home-based monitoring is for comparison or to track the individual's condition at certain times. If there's an abnormal result, they should immediately consult with a healthcare provider or the nearest facility. The hope is that there will be guidance from our personnel when they detect any issues with the patients, so that they don't rely solely on the application and skip their in-person check-ups. These check-ups involve more than just blood pressure; there are other examinations like measuring height, fundal height, and so on. (****IDI, health system manager****)*<br><br>• *I will hand it over to the midwife. So that what I said earlier doesn't happen, what's that, she has high blood pressure, low blood pressure, so she's really weak, that means she needs a health check. (****FGD, male CHW, older)****
**Changes in relationship**	• *I would be happy; I would be even happier if the patients are actively asking questions like that. Especially with complaints like "Midwife, I have this kind of problem." This way, we can*

Theme	Illustrative quotes
**with pregnant women**	*be more familiar with the patients. So, if we recommend something to the patient and they respond quickly, especially if there are complaints that we find that may lead to fatal pregnancy, for example, we can quickly respond. But if the patient remains silent and doesn't ask any questions, it takes longer for them to respond.* ***(IDI, facility midwife, older)***<br><br>• *The relationship will definitely be better, won't it? Because we can have effective two-way communication. We will find it difficult to make them unable to answer questions like "why" and "when". So far, the question is "when", and they can't answer. If they could take their own measurements, diagnostic accuracy would be better (****IDI, Ob/Gyn****)*
**Data use and exchange**	• *It's not a problem as long as we don't have to go to the field physically. So, we don't get tired physically. We just receive their health reports on the table. Unless the blood pressure is high, then we need to be alert and responsive. We will tell the patient to go to the health centre. For example, pregnant woman A says, "… I just measured my blood pressure. It turns out to be 150." - "In that case, go to the health centre immediately" usually, the patient will confide through WhatsApp regarding their blood pressure results. That's usually what we receive. And the staff must be ready to receive consultations at any time the patient wants. If it were me, no matter what time it is, I promise not to sleep.* ***(IDI, facility midwife, older)***<br>• *If it's with the midwife, at least once every two weeks or once a month. If we record the blood pressure, we can send it via WhatsApp so that she can respond. (****IDI, female CHW, older)***<br>• *I think it should be directly linked to the existing medical records because we also do some manual work, and the data we manually record is the basis for our entry into the e-Cohort system [digital pregnancy tracking system].* ***(IDI, health system manager)***
