# Supplementary material for: Health workers’ perspectives on self-monitoring of blood pressure by pregnant women: a qualitative study among community health workers, midwives, doctors and health system managers in Lombok, Indonesia
Source: BMJ Glob Health. 2025 Mar 22;10(3):e017532. doi: 10.1136/bmjgh-2024-017532 (PMC11931923; doi:10.1136/bmjgh-2024-017532)
Supplement: online supplemental file 2 [file bmjgh-10-3-s002.docx]

**Reflexivity statement**

The study author team consisted of maternal and digital health researchers based in Indonesia, as well as researchers from different geographic backgrounds and seniority levels, working at the global level or in academia. Three of the co-authors (AG, RMP, and OT) are non-practising clinicians, and another set of authors have obtained ANC services as pregnant women, two of whom have experienced HDP (MB, YDS, and TT). These experiences as service providers and ANC service-users may implicitly influence our interpretation of the data. We also acknowledge that our positions of working on global policies at the World Health Organization (OT, TT, MB) may make us particularly attuned to issues related to feasibility, equity and broader health system implications. This was balanced with perspectives from researchers and implementers working in Lombok to ensure that contextual nuances are not overlooked.
